# Primary Left Cardiac Angiosarcoma with Mitral Valve Involvement Accompanying Coronary Artery Disease

**DOI:** 10.1155/2015/810306

**Published:** 2015-11-16

**Authors:** Cagdas Baran, Serkan Durdu, Sadik Eryilmaz, Mustafa Sirlak, A. Ruchan Akar

**Affiliations:** Department of Cardiovascular Surgery, Ankara University School of Medicine, Cebeci Heart Center, Dikimevi, 06340 Ankara, Turkey

## Abstract

We report here on a 43-year-old female patient presenting with non-ST elevation myocardial infarction, severe mitral regurgitation, and mild mitral stenosis secondary to encroachment of the related structures by a primary cardiac angiosarcoma. A coronary angiography revealed significant stenosis in the left main and left circumflex arteries and at exploration, the tumour was arising from posterior left atrial free wall, invading the posterior mitral leaflet, and extending into all of the pulmonary veins and pericardium. Therefore, no further intervention was performed, except for left internal mammarian artery to left anterior descending artery anastomosis and biopsy. As far as we know, this case is unique with respect to its presentation.

## 1. Introduction

Primary cardiac tumours are rare clinical entities with a frequency of 0.0017 to 0.03% based on autopsies. Most of the primary cardiac tumours are benign, but approximately 25% are malignant, and the majority of these are sarcomas [1]. Angiosarcomas usually originate in the right atrium and are associated with a poor prognosis because of their aggressive nature and delay in the diagnosis due to insidious onset [2].

Herein, we report on a case of primary angiosarcoma of the left atrium. To the best of our knowledge, this case is unique in that the patient presented with mitral insufficiency and AMI (Acute Myocardial Infarction) due to encroachment of the related structures by the tumour.

## 2. Case Report

**Figure 1:**
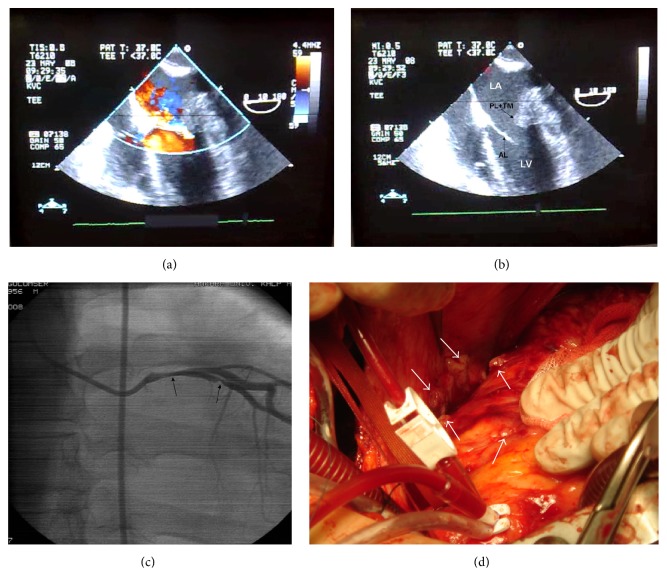
((a) and (b)) Transesophageal echocardiogram midesophageal 4-chamber view at 0°. (a) Color Doppler image shows severe (III/IV) mitral regurgitation. (b) The two-dimensional image shows an echogenic mass in the left atrium, arising from the region of the posterior mitral leaflet and causing restriction of the movements of this leaflet. (c) Coronary angiogram (left anterior oblique view with caudal angulation) showing significant stenoses at the left main (70%) and circumflex (80%) arteries (arrows). (d) Intraoperative view of the pericardial space. Extensive and multiple masses in the pericardium and multifocal central hemorrhage in the epicardium are shown.

A 43-year-old woman with no known history of CAD (Coronary Artery Disease) was presented to our hospital's cardiology clinic with a six-hour history of severe central chest pain and dyspnea. She does not have any known traditional CAD risk factors, arrhythmias, and was haemodynamically relatively stable at the time of admission. Cardiac auscultation revealed a grade 2-3/6 systolic murmur that was the loudest at the apex (fifth left intercostal space, midclavicular line). Lungs were clear to auscultation. Electrocardiography demonstrated sinus tachycardia (105/beats/minute) and ST-segment depression of approximately 3 mm in leads V1 to V6. Her cardiac markers were elevated (troponin I: 14.6 ng/L; normal range: 0.0–0.01 ng/L and creatinine kinase-MB mass: 53.9 ng/mL; normal range: 0.0–3.6 ng/mL). So a diagnosis of acute non-ST elevation myocardial infarction was made, and the patient was started on aspirin, intravenous heparin, intravenous nitroglycerin, and intravenous metoprolol. After TT (Transthoracic) and TE (Transesophageal) echocardiography, she was also diagnosed as having an echogenic mass in the left atrium arising from the region of the posterior mitral leaflet and causing restriction of the movement of this leaflet and, consequently, a grade III/IV mitral regurgitation and a mild degree of mitral stenosis (mean mitral valve area: 2.7 cm^2^ and mean transmitral diastolic gradient: 6 mmHg) (Figures 1(a) and 1(b)). The patient was then taken to the cardiac catheterization laboratory. Coronary angiogram revealed significant stenosis in the main coronary and left circumflex arteries (70% and 80% in diameter, resp.) and a normal right coronary artery (Figure 1(c)). Then, an informed consent was obtained and the patient underwent emergency open heart surgery. A surgical exploration revealed extensive and multiple solid masses in the pericardium and multifocal central hemorrhage on the epicardium (Figure 1(d)). These findings were to make it extremely difficult to isolate the coronary arteries, and therefore only a left interior mammarian artery to left anterior descending artery anastomosis was performed.

**Figure 2:**
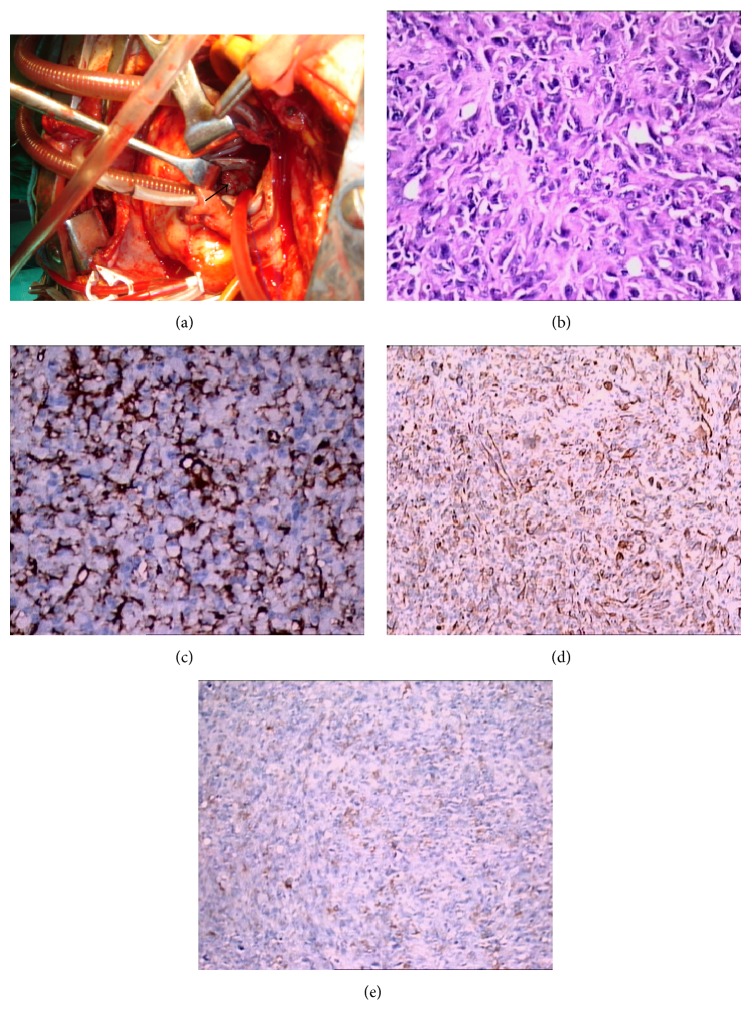
Intraoperative situs with view through the intra-atrial septum (transseptal approach). The arrow marks the roughly 1 cm large tumour originating at the posterior leaflet of the mitral valve (a). Haematoxylin and Eosin stain displaying a tumour consisting of irregular neoplastic vascular channels surrounded by atypical spindle shaped and epithelioid tumour cells (×40) (b). In immunohistochemical staining the tumour cells are stained with CD31 (×40) (c) and focally with Pan CK (×20) (d) and S-100 (×20) (e). The cells are pleomorphic and atypical.

After a right atriotomy with a transseptal incision/approach, a 1 × 1 cm lobulated solid mass, arising from the region of the posterior mitral leaflet in the posterior left atrial free wall and extending into all of the pulmonary veins, was observed (Figure 2(a)).

Because of the extension of the tumour, it was not possible to perform radical excision. We also did not attempt to debulk part of the tumour, since it was causing only a mild degree of mitral stenosis. Surprisingly, a final diagnosis of a highly differentiated angiosarcoma was made at the histopathological/immunohistochemical examinations (Figures 2(b), 2(c), 2(d), and 2(e)).

No metastasis was found by thoracoabdominal computerized tomography (CT) scans, brain magnetic resonance imaging (MRI), and bone scintigraphy. The patient was referred to the medical oncology department for possible chemotherapeutic and/or radiotherapeutic interventions.

The patient died because of heart failure at 12th week after cardiac surgery.

## 3. Discussion

In a study of 149 patients, the clinical symptoms of primary cardiac tumours were summarized as follows: (a) tumour mass that obstructs intracardiac blood flow or interferes with valve function, (b) arrhythmias or pericardial effusion with tamponade, (c) tumour embolism, and (d) systemic or constitutional symptoms [3]. Other presenting symptoms include haemoptysis secondary to diffuse pulmonary hemorrhage and other clinical features related to metastasis [4].

The patient described in this paper had a left atrial angiosarcoma, which has been extremely rarely reported in the literature [5]. To the best of our knowledge, there have only been two previous reports describing involvement of the mitral valve by an angiosarcoma [5]. In both of these case reports, the patients presented with severe functional mitral valve stenosis. There has also been a report documenting a patient with primary cardiac angiosarcoma involving the tricuspid valve and the RCA [6]. However, our patient is unique in that she presented with mitral regurgitation and AMI due to encroachment of the related structures by the tumour.

In the past, most angiosarcomas were diagnosed at postmortem examination. Modern echocardiography and high resolution tomographic imaging (CT, magnetic resonance tomography) have improved the diagnostic capabilities, facilitating an earlier diagnosis, which may result in an improved prognosis of this highly malignant neoplasm [7]. As seen in our case, echocardiography may be extremely useful in describing the morphology, size, and origin of a cardiac mass.

Based on the above-mentioned knowledge, it can be stated that an unexplained cardiac mass on echocardiography should prompt the clinician to continue to search for a malignant etiology using all diagnostic modalities including surgical exploration. However, even though various advanced diagnostic imaging modalities are applied, it might still be difficult to detect this malignancy at an early stage in the vast majority of the cases. Also in our case, the disease was in an advanced stage at the time of diagnosis. Additionally, there was no chance for us to perform some of the above-mentioned diagnostic modalities because of emergent status of the patient.

The prognosis of cardiac angiosarcoma is generally poor, with survival ranging from 6 to 12 months after the diagnosis has been established [8].

## 4. Conclusion

The present case underlines that a cardiac tumour, namely, primary angiosarcoma, may also cause acute coronary syndrome and/or mitral regurgitation by encroaching on the relevant structures.
